# Concurrently Affected by Dengue and Hepatitis A: Exploring the Intricacies of Co-infection in a Comprehensive Case Series

**DOI:** 10.7759/cureus.61863

**Published:** 2024-06-06

**Authors:** Keta Vagha, Punam Uke, Ashish Varma, Chaitanya Kumar Javvaji, Aashita Malik, Siddhartha Murhekar

**Affiliations:** 1 Pediatrics, Jawaharlal Nehru Medical College, Datta Meghe Institute of Higher Education and Research, Wardha, IND; 2 Pediatrics, Datta Meghe Medical College, Datta Meghe Institute of Higher Education and Research, Nagpur, IND; 3 Trauma and Orthopedics, East Kent Hospitals University NHS Foundation Trust, Canterbury, GBR

**Keywords:** dengue virus infection, hepatitis a virus (hav), co-infection, infectious disease, hepatits a, dengue

## Abstract

Based on the examination of four distinct cases, this case series offers a thorough investigation of the intricate relationship between dengue fever and hepatitis A infection. Despite their distinct origins, both illnesses manifest overlapping clinical features, posing considerable diagnostic hurdles, particularly in endemic regions. The cases reveal consistent symptoms such as elevated fever, abdominal discomfort, jaundice, and irregular liver function test results, underscoring the intricate nature of an accurate diagnosis. Variations in age distribution and the severity of symptoms underscore the necessity for tailored treatment approaches. Diagnostic challenges stem from the similarity in clinical presentations and shared laboratory abnormalities, necessitating comprehensive serological assessments. Therapeutic strategies entail a multidisciplinary approach addressing both hepatic and systemic manifestations, with supportive measures ensuring favorable clinical outcomes. Despite the complexities involved, timely interventions facilitate gradual symptom amelioration and successful patient recovery. Informing clinical practice and directing public health actions, this case series provides insightful information about the diagnostic and treatment complications associated with co-occurring dengue fever and hepatitis A infection.

## Introduction

In recent years, the co-infection of infectious diseases has become a topic of increasing concern within the realm of global public health. Among the myriad challenges faced by healthcare professionals, the simultaneous manifestation of hepatitis A infection and dengue fever presents a particularly complex and intriguing scenario [1]. Two different infectious illnesses that provide serious global public health issues are hepatitis A and dengue fever [2,3]. A thorough understanding of both their distinct traits and the complexities of their co-occurrence is necessary for efficient diagnosis, treatment plans, and preventative measures.

Dengue fever and hepatitis A are prominent infectious diseases, particularly in tropical and subtropical regions, where their endemicity poses significant public health challenges. Dengue fever, which is caused by the dengue virus and transmitted primarily through *Aedes aegypti* or *Aedes albopictus* mosquitoes, is characterized by a spectrum of clinical manifestations ranging from mild febrile illness to severe forms such as dengue hemorrhagic fever and dengue shock syndrome. Its clinical presentation often includes high fever, severe headache, retro-orbital pain, myalgia, arthralgia, rash, and, in severe cases, bleeding tendencies and organ impairment [4].

Hepatitis A, a positive-strand ribonucleic acid (RNA) virus, is an acute viral infection of the liver caused by the hepatitis A virus (HAV) and is primarily transmitted through the fecal-oral route, often via contaminated food or water. It typically results in a self-limiting illness with symptoms such as jaundice, anorexia, malaise, abdominal pain, and elevated liver enzymes. While generally less severe than other forms of viral hepatitis, hepatitis A can cause significant morbidity, particularly in adults [5].

The concurrent infection with dengue virus and HAV presents a unique clinical conundrum. The overlapping symptomatology, including fever, malaise, nausea, and elevated liver enzymes, can obscure the diagnosis and complicate the management. Moreover, the co-occurrence may exacerbate the severity of clinical manifestations, leading to increased morbidity and posing challenges for healthcare providers in regions where both infections are prevalent [6].

The co-occurrence of hepatitis A and dengue fever poses unique challenges in clinical management and public health response [1]. Patients presenting with symptoms suggestive of either disease may require comprehensive diagnostic evaluations to confirm the presence of both infections. Moreover, the management of concurrent infections necessitates a multidisciplinary approach, addressing both the hepatic and systemic manifestations of the diseases. In this detailed case series, we aim to explore the complexities of concurrent dengue fever and hepatitis A infection by describing four cases that showcase different presentations of this co-infection.

## Case presentation

Case 1

A 10-year-old boy was admitted to our hospital presenting with a high-grade intermittent fever persisting for 10 days, abdominal pain for four days, multiple episodes of vomiting over the past two days, and an icterus for two days. The fever was not associated with chills or rigors, and the abdominal pain was diffuse and dull. On examination, the patient was febrile (101 °F), with a heart rate (HR) of 112 beats per minute, a respiratory rate (RR) of 34 breaths per minute, and a blood pressure (BP) of 108/70 mmHg. There were no signs of dehydration. Abdominal examination revealed mild abdominal tenderness in the right hypochondrium, and the liver was palpable 2 cm below the costal margin (BCM); it was soft with a smooth surface.

The baseline blood investigations revealed a hemoglobin level (HB) of 12.7 g/dL, a total leukocyte count (TLC) of 5000/cubic millimeter (cumm), platelets at 184,000/cumm, and abnormal liver function tests (LFTs) showing alanine transaminase (ALT) at 1914 mg/dL, aspartate transaminase (AST) at 1888 mg/dL, alkaline phosphatase (ALP) at 392 mg/dL, and total bilirubin at 4.1 mg/dL (conjugated 3.2 mg/dL, unconjugated 0.9 mg/dL). The renal function tests and other blood parameters were within normal limits. Considering the regional prevalence of dengue fever, a dengue serology test was performed and revealed immunoglobulin M (IgM) positivity. Attributing the abnormal LFT results to dengue, no additional tests were initially conducted.

The patient was started on intravenous fluids in accordance with the dengue treatment protocol, and his vital signs were closely monitored. To assess the degree of hemoconcentration, his complete blood count was regularly checked, showing no further deterioration, such as a decrease in platelets or an increase in hematocrit levels. However, given the continued deterioration of LFT results, a co-infection was suspected, and an HAV serology was conducted, returning a positive result with an IgM value of 3.2 (positive > 1.2).

Consequently, a diagnosis of dengue fever with concurrent hepatitis A was made. Blood parameters were closely monitored, and supportive treatment was continued. From the third day of hospitalization, the patient exhibited clinical improvement, corroborated by improving laboratory values. He was discharged following a consistent decrease in the elevated liver enzyme levels and a satisfactory increase in platelet count.

Case 2

A 16-year-old boy was admitted to our hospital with a four-day history of fever, diffuse non-specific abdominal pain for two days, and increased work of breathing for one day. On examination, he was febrile at 102 °F, with tachycardia (HR of 122 beats per minute) and tachypnea (RR of 36 breaths per minute). His oxygen saturation on room air was 88%, and his BP was 112/70 mmHg. The systemic examination revealed reduced air entry bilaterally in the inframammary area without added sounds and a palpable liver extending 2 cm BCM with a soft consistency. No other systemic abnormalities were noted.

**Figure 1:**
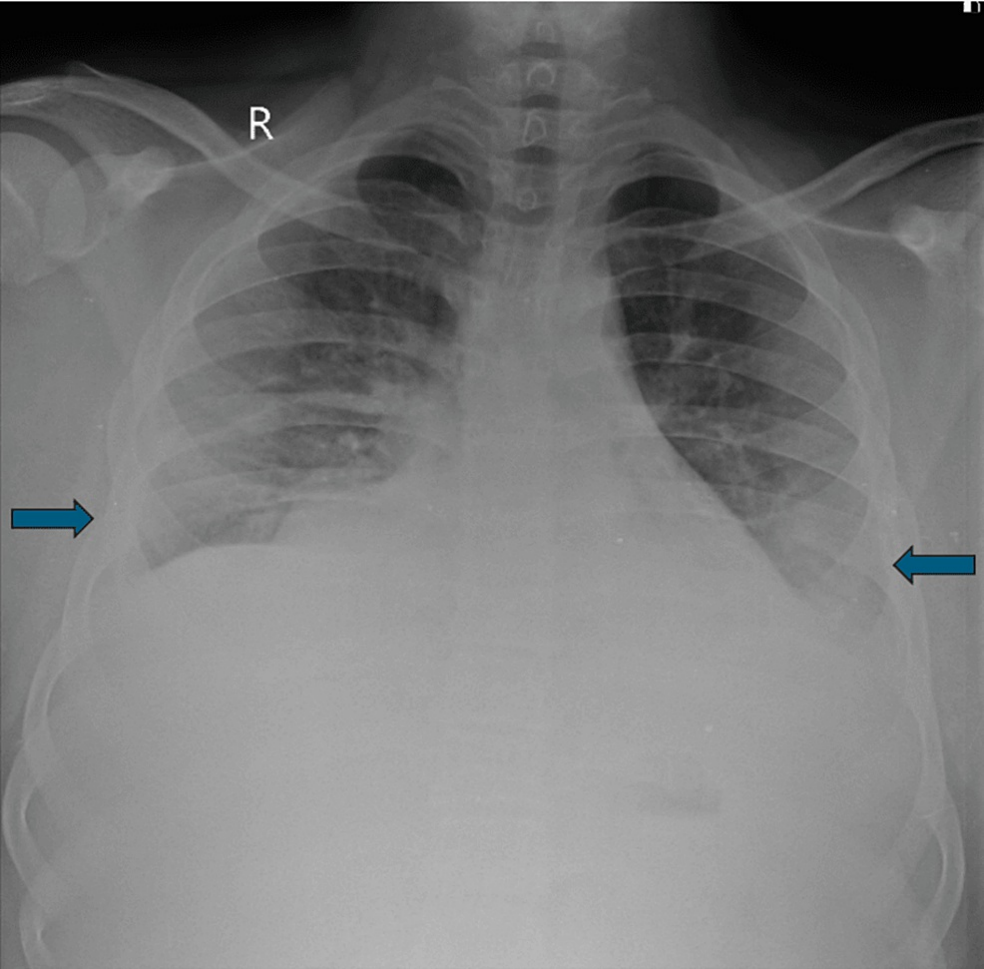
Chest radiograph of the patient showing bilateral pleural effusion denoted by the blue arrows.

The baseline blood investigations showed an HB level of 12.3 gm%, a TLC of 4500/cumm, and platelets at 90,000/cumm. LFTs were abnormal, with ALT at 1958 mg/dL, AST at 3506 mg/dL, ALP at 126 mg/dL, and total bilirubin at 1.5 mg/dL (conjugated: 0.7 mg/dL, unconjugated: 0.8 mg/dL). His coagulation profile showed a prothrombin time and international normalized ratio (PT-INR) of 1.99 and an activated partial thromboplastin time of 38.2 seconds. The dengue serology test was positive for the non-structural protein 1 (NS1) antigen, and he also tested positive for HAV-IgM, indicating co-infection with both viruses. A chest radiograph suggested mild bilateral pleural effusion (Figure 1).

The patient was started on oxygen supplementation via mask, intravenous fluids, antibiotics, and vitamin K to address the deranged coagulation profile. Following the initiation of treatment, the boy showed gradual improvement in clinical and laboratory parameters, allowing for his discharge after four days of hospitalization due to satisfactory progress.

Case 3

A five-year-old male child was admitted to our hospital presenting symptoms of fever persisting for 10 days and icterus for four days. The fever was characterized as high-grade and accompanied by chills and rigors. Upon examination, he presented as afebrile, with an HR of 92 beats per minute, RR of 20 breaths per minute, BP of 108/70 mmHg, and maintaining oxygen saturation at 94% on room air, with noticeable icterus in the sclera. The systemic examination revealed a palpable liver 4 cm BCM, with soft to firm consistency, and no other discernible systemic abnormalities. Two hours post-hospitalization, the patient experienced one episode of melena. The initial blood parameter analysis revealed an HB level of 10.4 g/dL, a TLC of 8100/cumm, platelet count of 98,000/cumm, and abnormal LFTs, indicating elevated levels of ALT at 362 mg/dL, AST at 178 mg/dL, ALP at 519 mg/dL, and total bilirubin at 7.2 mg/dL (conjugated 6.2 mg/dL, unconjugated 1.3 mg/dL). The renal function tests and other blood parameters remained within normal ranges. The dengue serology test returned positive for IgM; however, due to higher serum bilirubin levels than typically seen in dengue cases, HAV-IgM testing was initiated, yielding unexpectedly positive results with a titer of 3.2. The patient was commenced on supportive management, demonstrating gradual improvement over the subsequent three days, as evidenced by both clinical and laboratory observations.

Case 4

A 12-year-old female patient was admitted to our tertiary care hospital presenting symptoms of high-grade fever persisting for four days, accompanied by headaches for three days and episodes of vomiting for two days. The fever was characterized as high-grade and associated with chills and rigors. She reported typical symptoms of retro-orbital pain and photophobia. Upon examination, she presented as afebrile, with an HR of 82 beats per minute, RR of 16 breaths per minute, BP of 112/70 mmHg, and maintaining oxygen saturation at 92% on room air. The systemic examination revealed a palpable liver 2 cm BCM, with a soft consistency, and no other discernible systemic abnormalities. The initial blood parameter analysis revealed an HB level of 13.1 g/dL, a TLC of 4400/cumm, platelet count of 1.06 lacs/cumm, and abnormal LFTs, indicating elevated levels of ALT at 2019 mg/dL, AST at 1500 mg/dL, ALP at 395 mg/dL, and total bilirubin at 2.6 mg/dL (conjugated 1.8 mg/dL, unconjugated 0.8 mg/dL). The renal function tests and other blood parameters remained within normal ranges.

Considering the patient's history of fever with chills and rigors, and the presence of retro-orbital headaches, a dengue serology test was conducted, revealing positive IgM results. However, due to previous similar cases and the notably elevated liver enzymes, HAV-IgM testing was also performed, resulting in a positive finding with a titer of 3.6, marking the fourth case of such co-infection. The patient was initiated on supportive management, comprising intravenous fluids, antibiotics, and antipyretics. Over the subsequent four days, she exhibited gradual improvement, as observed both clinically and through laboratory parameters.

The sequential and comprehensive laboratory values of all four patients are meticulously documented in Table 1.

**Table 1:** Sequential laboratory parameters of all four patients. TLC: total leucocyte count; HCT: hematocrit; ALT: alanine transferase; AST: aspartate transferase; ALP: alkaline phosphatase; PT-INR: prothrombin time-international normalized ratio; APTT: activated partial thromboplastin time; M: male; F: female; IgM: immunoglobulin M; NS1: non-structural protein-1; HAV: hepatitis A virus; (-): investigations not done.

Investigations	Case 1					Case 2				Case 3			Case 4				Normal values with units
	Day 1	Day 2	Day 3	Day 4	Day 5	Day 1	Day 2	Day 3	Day 4	Day 1	Day 2	Day 3	Day 1	Day 2	Day 3	Day 4	
Hemoglobin	12.7	12.8	11.8	11	-	12.3	11.9	11.1	12	10.4	9.4	-	13.1	12.1	-	12.7	11-14 g/dL
TLC	5000	7100	11,300	6600	-	4500	8700	8500	9200	8100	6000	-	4400	3500	-	4400	5000-15,000 cumm
Platelets	1.84	1.9	1.83	1.99	-	90,000	1.17	1.35	1.4	98,000	1.19	-	1.06	1.03	-	2.43	2.0-4.9 lacs/cumm
HCT	38.5	38.9	35.9	34.1	-	37.1	35.5	33.7	35.5	31.9	28.8	-	38.9	35.9	-	37.5	34-40%
Total bilirubin	4.1	-	3.4	-	1.6	1.5	1.6	-	1.1	7.5	-	3.3	2.6	-	4	3.6	0.2-1.3 mg/dL
Conjugated	3.2	-	2.8	-	1.1	0.7	0.5	-	0.2	6.2	-	2.6	1.8	-	3	2.7	0.0-0.3 mg/dL
Unconjugated	0.9	-	0.6	-	0.5	0.8	1.1	-	0.9	1.3	-	0.7	0.8	-	1	0.9	0.0-1.1 mg/dL
ALT	1914	-	936	-	557	1958	1275	-	466	362	-	199	2019	1622	750	595	M: <50 U/L; F: <35 U/L
AST	1888	-	417	-	212	3506	1415	-	156	178	-	222	1500	1142	597	333	M: 17-59 U/L; F: 14-36 U/L
ALP	392	-	435	-	391	126	129	-	139	519	-	716	395	-	392	366	38-126 U/L
Total protein	7.1	-	6.5	-	7.8	6.3	6.2	-	8	6.6	-	7.5	7.5	-	8.3	8.3	6.3-8.2 g/dL
Albumin	3.5	-	3.1	-	3.7	2.8	2.7	-	3.6	3.1	-	3.4	3.9	-	4	3.9	3.5-5.0 g/dL
Globulin	3.6	-	3.4	-	4.1	3.5	3.5	-	4.4	3.5	-	4.1	3.6	-	4.3	4.4	2.3-3.5 g/dL
Urea	17	-	-	-	-	23	-	-	10	20	-	-	19	-	-	-	M: 9-20 mg/dL; F: 7-17 mg/dL
Creatinine	0.7	-	-	-	-	0.9	-	-	0.6	0.6	-	-	0.6	-	-	-	M: 0.66-1.25 mg/dL; F: 0.52-1.04 mg/dL
Sodium	139	-	-	-	-	139	-	-	142	136	-	-	137	-	-	-	137-145 mmol/L
Potassium	4.7	-	-	-	-	3.9	-	-	3.9	4.8	-	-	4	-	-	-	3.5-5.1 mmol/L
PT-INR	1.17	-	-	-	-	1.99	-	-	1.27	1.26	-	-	1.22	1.29	-	1	1-1.3
APTT	38	-	-	-	-	38.2	-	-	36.3	45.1	-	-	34.4	36.8	-	36.5	29.5 sec
Dengue serology	IgM positive					NS1 antigen positive				IgM positive			IgM positive				-
HAV-IgM	11.7					2.8				3.2			3.6				>1.20

## Discussion

Hepatitis A and dengue fever are caused by distinct viruses with different modes of transmission and pathophysiological mechanisms. The single-stranded RNA virus that causes hepatitis A is a member of the *Picornaviridae *family of viruses. Hepatocytes are the primary target of this virus, which causes inflammation and malfunction in the liver. Usually, contaminated food or water is the means by which HAV is spread through the fecal-oral pathway [7]. On the other hand, the dengue virus, a flavivirus mainly spread by *Aedes* mosquitoes, is what causes dengue fever. Dendritic cells, macrophages, and monocytes are the main targets of the virus, which has four different serotypes. In extreme cases, dengue fever can cause hemorrhagic symptoms, thrombocytopenia, and vascular leakage as a result of a systemic inflammatory response [8].

Hepatitis A is very common worldwide, particularly in communities with inadequate sanitation and hygiene standards. Although vaccination campaigns and better sanitation have reduced the prevalence in many affluent nations, outbreaks sometimes happen, particularly in unsanitary areas [9]. Visiting endemic areas, living in unsanitary conditions, and overcrowding are risk factors for contracting HAV infection. Dengue fever is widespread in more than 100 countries, mainly in tropical and subtropical areas. The number of dengue cases has surged in recent decades, with approximately 390 million infections each year [10]. Factors contributing to the risk of dengue infection include urbanization, population growth, and climate change, all of which affect the distribution and population of mosquito vectors.

Hepatitis A usually starts with a prodrome of general symptoms such as tiredness, discomfort, loss of appetite, and queasiness, before progressing to jaundice, dark urine, and abdominal pain. The illness is usually self-limiting, with symptoms resolving within a few weeks. However, in rare cases, particularly in older adults or individuals with underlying liver disease, severe hepatitis and acute liver failure can occur [5]. Rapidly rising temperature, severe headache, discomfort behind the eyes, aches in the muscles and joints, and skin rash are the symptoms of dengue fever. People may also have symptoms such as nausea, vomiting, and stomach pain in addition to fever. Severe instances can develop into dengue shock syndrome, which is characterized by low platelet counts, bleeding difficulties, fluid leakage, or dengue hemorrhagic fever [11].

The co-infection of hepatitis A and dengue fever poses unique challenges due to non-differential clinical features and shared risk factors [1]. Despite their distinct etiologies and modes of transmission, hepatitis A and dengue fever share overlapping clinical features, which can complicate diagnosis, particularly in regions where both diseases are endemic [12]. Both illnesses commonly present with fever, malaise, and gastrointestinal symptoms, making it challenging to differentiate between them based solely on clinical grounds. Additionally, both diseases can manifest with jaundice, although it is more commonly associated with hepatitis A.

Epidemiologically, hepatitis A is more prevalent in areas with poor sanitation and hygiene practices, while dengue fever thrives in regions with a high density of *Aedes* mosquitoes. However, overlapping epidemiological factors, such as urbanization, population movement, and climate change, can contribute to the concurrent occurrence of both diseases in certain geographical areas [13]. The coexistence of dengue fever with other infections such as leptospirosis, hepatitis A, hepatitis E, malaria, or typhoid fever can complicate diagnosis and management due to overlapping clinical features [14].

Dengue infections often present with hepatomegaly, mild-to-moderate increases in transaminase levels, hemoconcentration, and third spacing, leading to ascites and pleural effusion. Jaundice in dengue can mimic acute hepatitis, making differentiation from hepatitis A crucial. Dengue-related liver involvement can result from direct viral effects, immune responses, or localized vascular leakage. Viral hepatitis typically shows serum aminotransferase levels 8-10 times normal, whereas dengue shows 2-3 times normal, with an AST/LDH ratio of >4 in viral hepatitis. Dengue features include hemoconcentration, thrombocytopenia, and third-space fluid losses. However, cases with fever and serositis but without hemoconcentration or thrombocytopenia suggest possible coexistent viral hepatitis, especially with abnormal coagulation profiles. Prolonged fever, highly elevated liver enzymes, deranged prothrombin time, and positive IgM for both hepatitis and dengue indicate possible co-infection [15].

This case series offers valuable insights into the interaction between hepatitis A infection and dengue fever. Common clinical features, including abdominal pain, high-grade fever, jaundice, and abnormal LFTs, were observed across all cases, highlighting diagnostic complexities. Variability in age distribution and severity of illness underscored the need for individualized patient care. Diagnostic challenges stemmed from overlapping clinical features and laboratory abnormalities, necessitating additional serological testing for confirmation. Therapeutically, a multifaceted approach addressing hepatic and systemic manifestations was crucial, with supportive measures and close monitoring ensuring favorable clinical outcomes. Despite complexities, appropriate management led to gradual symptom improvement and successful hospital discharge for all patients.

## Conclusions

In conclusion, the simultaneous occurrence of dengue fever and hepatitis A poses a diagnostic and treatment challenge for healthcare professionals. It is crucial to comprehend the distinct aspects of each infection and recognize their shared clinical traits to ensure precise diagnosis and effective management. Thorough analyses of case series, such as this one, provide a valuable understanding of the epidemiology, underlying mechanisms, and clinical strategies for managing these co-infections. Such insights are instrumental in shaping public health initiatives aimed at alleviating their impact. Additionally, including further testing, such as viral PCRs, in cases of co-infections could enhance diagnostic accuracy and guide more targeted therapeutic approaches, thereby improving patient outcomes.
